# Feasibility of Dedicated Breast Positron Emission Tomography Image Denoising Using a Residual Neural Network

**DOI:** 10.22038/AOJNMB.2023.71598.1501

**Published:** 2023

**Authors:** Koji Itagaki, Kanae K. Miyake, Minori Tanoue, Tae Oishi, Masako Kataoka, Masahiro Kawashima, Masakazu Toi, Yuji Nakamoto

**Affiliations:** 1 Division of Clinical Radiology Service, Kyoto University Hospital, Kyoto, Japan; 2 Department of Advanced Medical Imaging Research, Graduate School of Medicine Kyoto University, Kyoto , Japan; 3 Department of Diagnostic Imaging and Nuclear Medicine, Graduate School of Medicine Kyoto University, Kyoto, Japan; 4 Department of Breast Surgery, Graduate School of Medicine Kyoto University, Kyoto, Japan

**Keywords:** Deep neural networks, db PET, Noise reduction, Image quality

## Abstract

**Objective(s):**

This study aimed to create a deep learning (DL)-based denoising model using a residual neural network (Res-Net) trained to reduce noise in ring-type dedicated breast positron emission tomography (dbPET) images acquired in about half the emission time, and to evaluate the feasibility and the effectiveness of the model in terms of its noise reduction performance and preservation of quantitative values compared to conventional post-image filtering techniques.

**Methods:**

Low-count (LC) and full-count (FC) PET images with acquisition durations of 3 and 7 minutes, respectively, were reconstructed. A Res-Net was trained to create a noise reduction model using fifteen patients’ data. The inputs to the network were LC images and its outputs were denoised PET (LC + DL) images, which should resemble FC images. To evaluate the LC + DL images, Gaussian and non-local mean (NLM) filters were applied to the LC images (LC + Gaussian and LC + NLM, respectively). To create reference images, a Gaussian filter was applied to the FC images (FC + Gaussian). The usefulness of our denoising model was objectively and visually evaluated using test data set of thirteen patients. The coefficient of variation (CV) of background fibroglandular tissue or fat tissue were measured to evaluate the performance of the noise reduction. The SUV_max_ and SUV_peak_ of lesions were also measured. The agreement of the SUV measurements was evaluated by Bland–Altman plots.

**Results:**

The CV of background fibroglandular tissue in the LC + DL images was significantly lower (9.10±2.76) than the CVs in the LC (13.60± 3.66) and LC + Gaussian images (11.51± 3.56). No significant difference was observed in both SUV_max_ and SUV_peak_ of lesions between LC + DL and reference images. For the visual assessment, the smoothness rating for the LC + DL images was significantly better than that for the other images except for the reference images.

**Conclusion:**

Our model reduced the noise in dbPET images acquired in about half the emission time while preserving quantitative values of lesions. This study demonstrates that machine learning is feasible and potentially performs better than conventional post-image filtering in dbPET denoising.

## Introduction

 Malignant tumors are often detected and staged using [^18^F]fluorodeoxyglucose ([^18^F]FDG) positron emission tomography (PET) (1). [^18^F]FDG PET is also used for staging or re-staging breast cancer as well as predicting the response to therapy and diagnosing recurrence in breast cancer patients (2-5). However, whole body PET (WB-PET) may have a reduced ability to detect small (<10 mm) breast cancer tumors (6, 7). A recent study also reported that the sensitivity of dbPET was higher for tumors smaller than 10 mm compared to WB-PET (8). In addition, partial volume effects (PVE) in PET image occurs whenever the tumor size is less than three times the full width half maximum (FWHM) of the spatial resolution (9). The FWHM of the spatial resolution of commercially available WB-PET systems is reported to be 4.0-5.0 mm (10, 11). Because of the limited spatial resolution of WB-PET, the quantitative values of small tumors are affected by PVE (12).

 To overcome those limitations, high-resolution breast PET scanners have been developed. These systems have been used to detect breast cancer lesions, diagnose intra-mammary spread, assess the morphological details of tumors, and metabolic information (13). There are two types of high-resolution breast PET scanners: positron emission mammography (PEM) (14) and ring-type dedicated breast PET (dbPET) (15). PEM provides limited-angle tomographic images using two planar or curved detectors, whereas dbPET provides fully tomographic images of the breast with a ring-shaped detector (16). dbPET can provide PET images with higher spatial resolution than WB-PET because of the small size of the crystals, the proximity of the detectors to the breast and the reduction of non-collinearity effects due to smaller ring diameters. Miyake et al reported that the FWHM of the spatial resolution of the dbPET system is 0.8-1.3 mm when reconstructed with a clinically used reconstruction method (17). Because of the high spatial resolution of dbPET, it has been reported that the ability of dbPET to detect breast cancers smaller than 10 mm is better than that of WB-PET (8). A phantom study using microspheres less than 10 mm in diameter has also reported higher detectability with dbPET compared to WB-PET (18). In addition, Berg et al. previously reported that PEM had improved specificity compared with MRI (19). Furthermore, the usefulness of dbPET for evaluating the breast cancer response to neoadjuvant chemotherapy using the standardized uptake value (SUV) has also been reported (20).

 Despite the high maximal sensitivity at the center of the axial field of view of the dbPET system, the dbPET images often have a high level of noise, especially around the edge of the detector, due to a decrease in effective counts near the edge (16). A reconstruction method that prioritizes improvement of specificity of detected uptake patterns can also increase image noise. The noise in the dbPET images may lead to the detection of a larger number of non-pathologic uptake foci, and result in false-positive diagnoses (21). Several methods can be used to suppress the noise of PET images, including increasing acquisition time, post-image filtering, such as a Gaussian filter or non-local mean (NLM) filter (22), and applying Bayesian penalized likelihood reconstruction algorithms (23). Longer scan times increase the probability of motion artifacts and physical burden on a patient, especially in dbPET, which scans the patient in a prone position. A Gaussian filter is sometimes used for dbPET image denoising, but it can remove details of the tumor structure (24). An NLM filter can reduce image noise while preserving image details, but it requires some parameters to be optimized. Bayesian penalized likelihood reconstruction algorithms also need a regularization parameter to be set to control noise and preserve edges, and a bad optimization of this parameter leads to over-smoothed images.

 Recently, machine learning methods for PET denoising, such as those based on convolutional neural networks and U-Net (25, 26), have achieved improvements in both objective and subjective assessment. However, to our knowledge, no study has used machine learning for noise reduction in dbPET images, which have a higher spatial resolution than WB-PET. U-Net has occasionally been used for PET image denoising, but this architecture may cause blurred images due to the down-sampling and up-sampling, despite the use of skip connections (25-27). Blur in images is a problem, especially for dbPET, which requires high spatial resolution. Convolution filters with large kernel size increase the receptive field size without down-sampling and up-sampling, thus avoid blurring (28). A residual neural network (Res-Net) also prevents the blurring of images in machine learning-based denoising (29, 30).

 In this study, we created a deep learning (DL)-based denoising model using a Res-Net with large kernel size of convolution filters that was trained to reduce noise in dbPET images acquired in about half the emission time. We evaluated the usefulness of the model in terms of its noise reduction performance and preservation of quantitative values by comparing it with conventional post-image filtering.

## Materials

***Patient data***

 A total of twenty-eight consecutive patients with known or suspected breast cancers who underwent dbPET scan from February 2021 to December 2021 were included in this study. Patients fasted at least 4 h prior to administration of [^18^F]FDG (3.5 MBq/kg) and were scanned 90 minutes after administration. PET data were acquired in three-dimensional (3D) list mode for 7 minutes per breast using a dbPET scanner (Elmammo Avant Class, Shimadzu Corp., Kyoto, Japan). We also acquired 3 minutes of PET data from the list data. Low-count (LC) and full-count (FC) PET images with acquisition durations of 3 and 7 minutes, respectively, were reconstructed with the 3D list mode dynamic row-action maximum-likelihood algorithm (DRAMA) using one iteration, 128 subsets, and a relaxation control parameter of β =20 for all data sets. No post-smoothing was applied to the PET images. The matrix size was 236×132 with pixel sizes of 0.78×0.78 mm, and the slice thickness was 0.78 mm. Scatter correction was conducted using the convolution-subtraction method. Attenuation correction was performed using calculated uniform attenuation maps created from tissue boundaries estimated from the emission data. This study was performed in line with the principles of the Declaration of Helsinki. Approval was granted by the Ethics Committee of Kyoto University Graduate School and Faculty of Medicine (Approval number, R3034). Informed consent was waived by the Ethics Committee of Kyoto University Graduate School and Faculty of Medicine due to the retrospective design.

***Network architecture***

**Figure 1:**
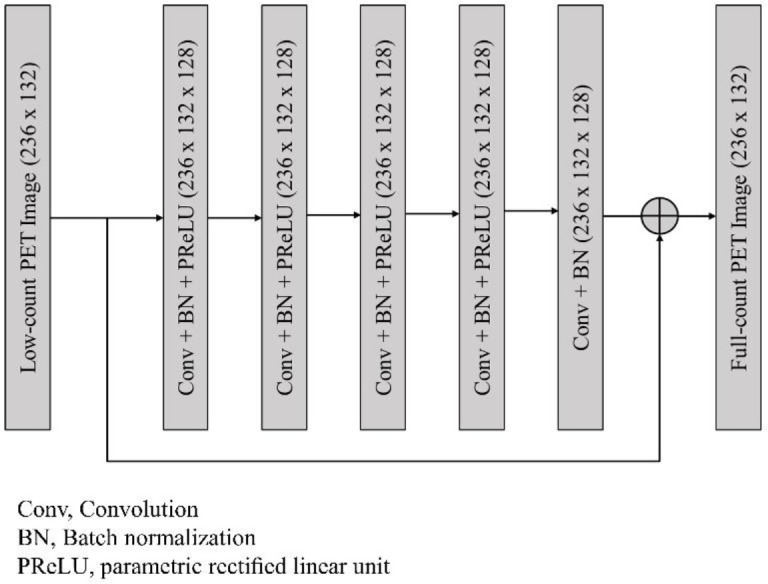
Network architecture employed in this study. The network was composed of five convolutional layers with a 15×15 kernel size, batch normalization (BN), and parametric rectified linear unit (PReLU)

 Our network structure was similar to the Res-Net used in a prior study (30). Skip connection from input-end to output-end was used in this architecture to compensate the lost details and to perform residual learning simultaneously. The network architecture is shown in Figure 1 and is composed of convolutional layers with a 15×15 kernel size, batch normalization (BN) (31), and parametric rectified linear unit (PReLU) activation (32). BN was added between the convolution and PReLU layers. The number of filters for each convolutional layer was 128, and the spatial size of the network input was 236×132. A larger receptive field size can make use of context information in a larger image region, and hence we adopted the large kernel size of 15×15. To avoid the problem of vanishing gradients that occurs the rectified linear unit (ReLU) activation function, we used PReLU as the activation function of the network.

***Network training***

 Fifteen patients’ data were used for training (6,372 images) and validation (708 images). We randomly reserved 10% of the training samples for validation data to monitor the performance of the network during training. The inputs for the network were two-dimensional (2D) LC images. The outputs were denoised 2D PET (LC + DL) images, which should resemble the FC images. The network was trained for 100 epochs and optimized using the Adam optimizer (33) to minimize the mean squared error (MSE). The batch size was 16, and a learning rate of 0.001 was used. The network was implemented using Keras with a TensorFlow backend (Google, Mountain View, California), and trained used a single NVIDIA GeForce RTX 2080Ti GPU (NVIDIA Corporation, Santa Clara, California).

***Comparison with conventional post-image filtering***

 To evaluate the incremental value of our denoising model, LC + DL images were compared to dbPET images denoised by conventional post-image filtering. Gaussian filter and an NLM filter were applied to the LC images (LC+Gaussian and LC+NLM, respectively). To create reference images, a Gaussian filter was also applied to the FC images to obtain the same reconstruction conditions as in our clinical setting (FC+Gaussian). The full width at half maximum of the Gaussian filter was 1.17 mm. For the NLM method, the patch size was 3×3, and the search window size was 5×5. The standard deviation of the Gaussian kernel used in the NLM method was set to be the standard deviation of the background fibroglandular tissue in breasts measured with the LC images. The other parameters of the NLM filter were determined based on previous reports (34, 35).

***Quantitative analysis***

 The performance of the noise reduction model was objectively evaluated using a test data set of thirteen patients with and without breast lesions. The SUV_mean_ and coefficient of variation (CV) of the background fibroglandular tissue without lesions were measured to assess the noise level of the images. In addition, the CV of the background fibroglandular tissue or fat tissue at the edge of the FOV was also measured. For visually FDG-avid breast lesions that were histologically confirmed or highly suspicious on imaging modalities other than dbPET, the SUV_max_ and SUV_peak_ were measured to evaluate the effect of this model on the lesion uptake values.

**Figure 2:**
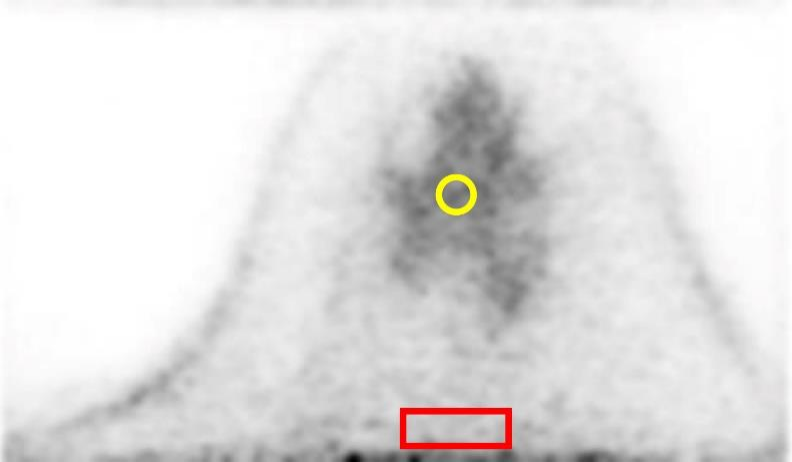
Placement of regions of interest (ROI) in the measurement of dbPET images. The SUV_mean_ and CV of the background fibroglandular tissue were obtained from five 2D-ROIs with a diameter of 8 mm (**yellow**) and the CV of the background fibroglandular tissue or fat tissue at the edge of the FOV were obtained from five 2D-ROIs of 10×30 pixel rectangles placed at 5 pixels from the FOV edge (**red**). Each ROI was placed in 5 different slices, which are at least 5 slices apart from each other to include as wide range of background fibroglandular tissue as possible

 The SUV_mean_ and CV of the background fibroglandular tissue were obtained from five 2D regions of interest (2D-ROIs) with a diameter of 8 mm per breast placed on background fibroglandular tissue. The CV of the background fibroglandular tissue or fat tissue at the edge of the FOV were obtained from five 2D-ROIs of 10×30 pixel rectangles placed at 5 pixels from the FOV edge. Each ROI was placed in 5 different slices, which are at least 5 slices apart from each other to include as wide range of background fibroglandular tissue or fat tissue as possible (Figure 2). The SUV_mean_ is an average of SUV within the 2D-ROI, and the CV was calculated using the following equation.

$\mathrm{CV}=\frac{\sigma }{{\mathrm{SUV}}_{\mathrm{mean}}}\times 100(\%)$

 Here, σ is the standard deviation (SD) within the 2D-ROI.

 The SUV_max_ of the lesions was obtained from a 3D volume of interest (3D-VOI). The SUV_peak_ was defined as the average SUV, which was measured in a 2D-ROI with a fixed diameter of 10 mm centered at the maximum value of the lesions. In addition, the agreement of the SUV measurements of the reference (FC + Gaussian) and each target image (LC, LC + Gaussian, LC + NLM, and LC + DL) was assessed. Relative differences were calculated for the SUV_max_ and SUV_peak_ using the FC + Gaussian images as reference, and the agreement was evaluated using Bland–Altman plots. The relative difference (d) between the reference and the target images was defined as the following equation.

$d=\frac{\left({\mathrm{SUV}}_{\mathrm{tgt}}-{\mathrm{SUV}}_{\mathrm{ref}}\right)}{{\mathrm{SUV}}_{\mathrm{ref}}}\times 100(\%)$

 Here, ${\mathrm{SUV}}_{\mathrm{tgt}}$and${\mathrm{SUV}}_{\mathrm{ref}}$are the SUV measurements obtained in the target and reference images, respectively. The bias and variance of the relative differences in the SUV measurements were defined as the mean and 1.96×SD of d, respectively.

 The lesions were classified into three types of uptake: focus, mass uptake (MU), and non-mass uptake (NMU), based on the 3D morphologic features with reference to a previous report (15).

 All analyses were performed using MATLAB 2021a (The MathWorks, Inc., Natick, Massa-chusetts).

***Visual assessment***

 For the visual assessment, craniocaudal (CC) and mediolateral (ML) maximum intensity projection (MIP) images of test data sets were visually evaluated for smoothness (degree of the image noise) and lesion contrast between the mammary gland and lesions using a four-point scale (0, not acceptable for diagnosis; 1, acceptable; 2, good; and 3, excellent) by an experienced nuclear medicine physician and an experienced PET technologist blinded to the reconstruction settings. For lesion contrast, FDG-avid breast lesions that were histologically confirmed or highly suspicious on imaging modalities other than dbPET were visually evaluated on CC or ML MIP images. The MIP images were displayed on an inverse grayscale with a standardized uptake range of 0–4.

***Statistical analysis***

 The SUV_mean_ and CV of background fibro-glandular tissue or fat tissue obtained for all image sets were compared using the paired t-test with Bonferroni correction. The SUV_max_ and SUV_peak_ of the lesions in the reference images and target images were compared using the Wilcoxon signed rank test with Bonferroni correction. Differences in patient characteristics in the data sets was examined using the Mann–Whitney U test and Fisher's exact test. Visual scores for all image sets were compared using the Wilcoxon paired ranked-sum test with Bonferroni correction. Inter-reader agreement was evaluated using Cohen’s kappa test. A p value of less than 0.05 was considered statistically significant for each analysis. The statistical analysis was performed using JMP® 16.1.0 (SAS Institute Inc., Cary, NC, USA).

## Results

Table 1 summarizes the characteristics of fifteen patients (age range, 34–81 years; mean age, 65 years; number of lesions, 20) in the training and validation data sets and thirteen patients (age range, 49–82 years; mean age, 63 years; number of lesions, 22) in the test data set. There was no significant difference between the two sets in terms of age and treatment prior to dbPET examinations.

**Table 1:** Characteristics of the enrolled patients

**Characteristics**	**Training + validation (n=15)**	**Test (n=13)**	***p *** **value**
Age (mean±SD)	65±14.5	63±11.6	0.53
Number of lesions	20	22	
Treatment prior to dbPET			1.00
No treatment	12	11	
Neoadjuvant chemotherapy	2	1	
Surgery	1	1	

**Figure 3:**
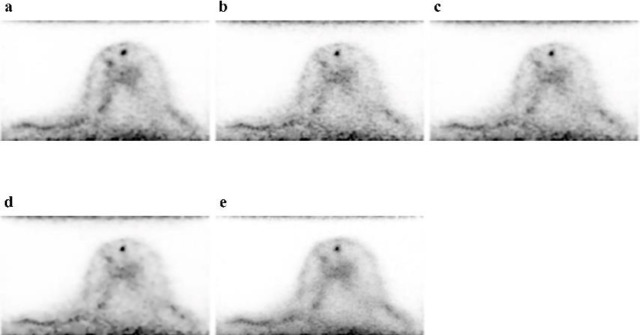
Trans-axial images of the reference (FC + Gaussian) image (**a**) and LC (**b**), LC + Gaussian (**c**), LC + NLM (**d**), and LC + DL (**e**) target images. The LC + DL image has lower noise levels than the LC image

**Figure 4:**
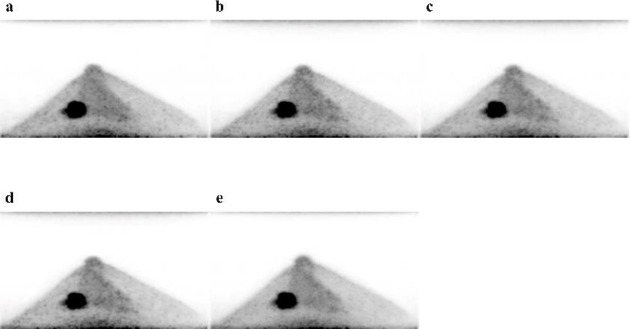
Craniocaudal maximum intensity projection images of the reference (FC + Gaussian) image (**a**) and LC (**b**), LC + Gaussian (**c**), LC + NLM (**d**), and LC + DL (**e**) target images of a 52-year-old woman with invasive ductal carcinoma in the left breast. The LC + DL image has noise levels that are visually lower than those in the other images

**Figure 5:**
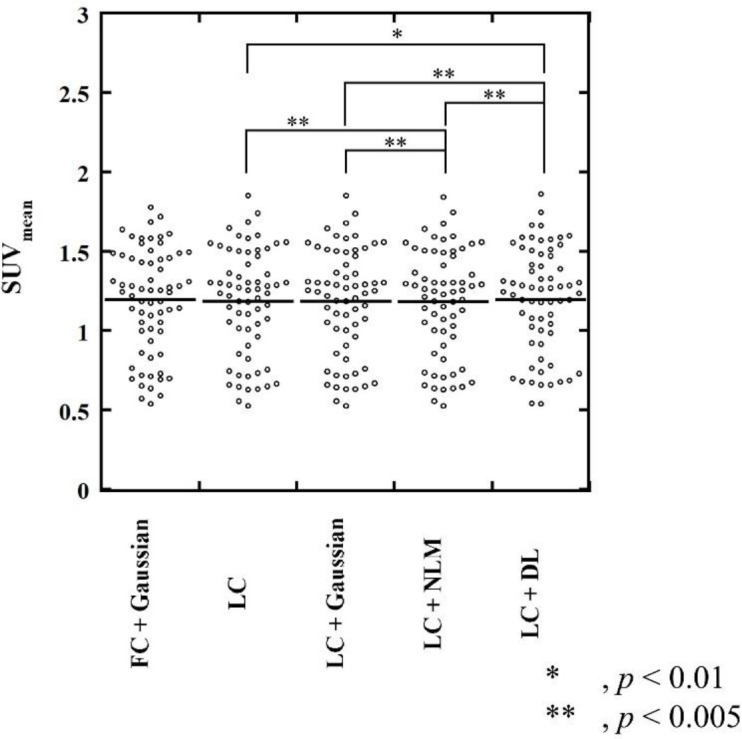
SUV_mean_ of background fibroglandular tissue. The SUV_mean_ of the background fibroglandular tissue in the LC + DL images was slightly higher than that in the LC, LC + Gaussian, and LC + NLM images, whereas no significant difference in SUV_mean _was observed between the LC + DL and the reference (FC + Gaussian) images

**Figure 6:**
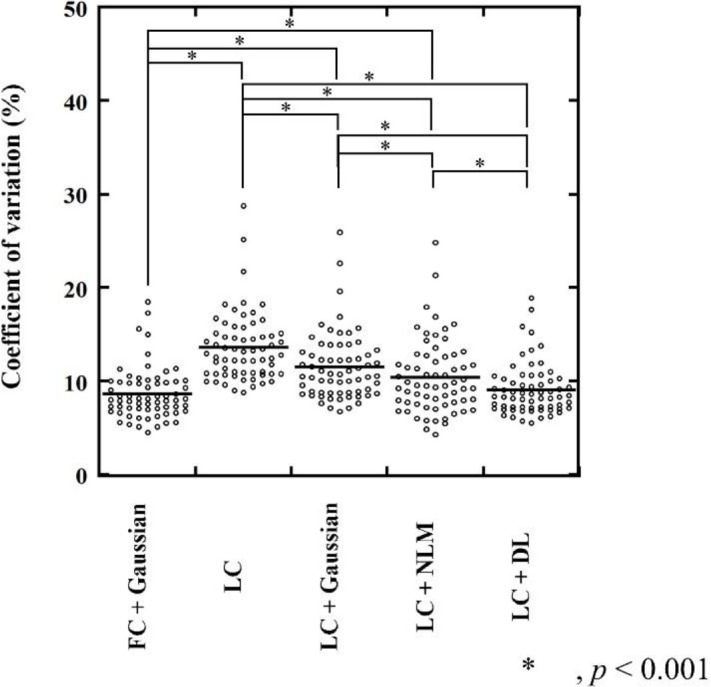
CV of background fibroglandular tissue. The CV of the background fibroglandular tissue in the LC + DL images was significantly lower than that in the LC, LC + Gaussian, and LC + NLM images (*p*<0.001), whereas no significant difference in CV was observed between the LC + DL and the reference (FC + Gaussian) images

**Figure 7:**
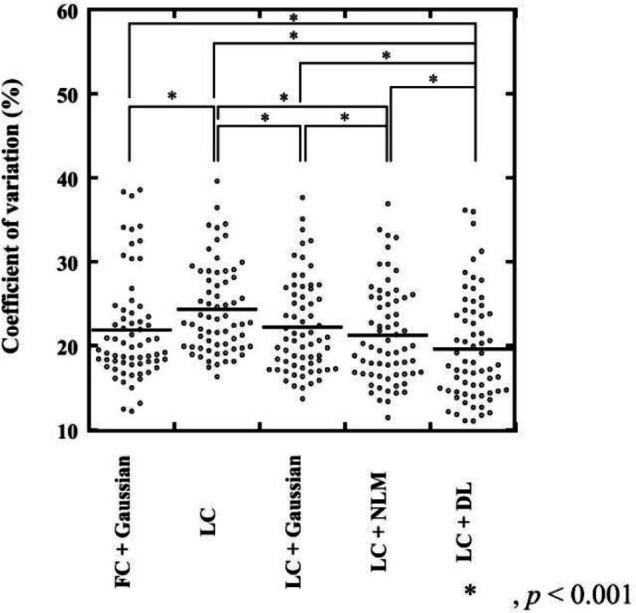
CV of the background fibroglandular tissue or fat tissue at the edge of the FOV. The CV of the background fibroglandular tissue or fat tissue at the edge of the FOV in the LC + DL images was significantly lower than that in the LC, LC + Gaussian, LC + NLM, and reference (FC + Gaussian) images (*p*<0.001)

For the reference and each of the target images, Figure 3 shows trans-axial images and Figure 4 shows CC MIP images. A subjective visual inspection revealed that the LC + DL image has lower noise levels than the LC image. The SUV_mean_ of the background fibroglandular tissue in the LC + DL images was slightly higher (mean±SD [95%confidence interval (CI)], 1.18±0.33 [1.11–1.27]) than that in the LC, LC + Gaussian, and LC + NLM images (mean±SD [95% CI], 1.18±0.34 [1.10-1.27], 1.18±0.34 [1.10–1.27] and 1.18±0.33 [1.10-1.26] respectively), whereas no significant difference in SUV_mean_ was observed between the LC + DL and reference images (mean±SD [95% CI], 1.19±0.33 [1.11-1.28]). The CV of the background fibroglandular tissue in the LC + DL images was significantly lower (mean±SD [95% CI], 9.10±2.76 [8.42–9.79]) than that in the LC, LC + Gaussian, and LC + NLM images (mean±SD [95% CI], 13.60±3.66 [12.69–14.51], 11.51±3.56 [10.63–12.39]and 10.40±3.92 [9.43–11.38], respectively), whereas no significant difference in CV was observed between the LC + DL and reference images (mean±SD [95% CI], 8.64±2.75 [7.96–9.32]). Figure 5 and Figure 6 show the SUV_mean_ and CV of the background fibro-glandular tissue. The CV of the background fibroglandular tissue or fat tissue at the edge of the FOV in the LC + DL images was significantly lower (mean±SD [95% CI], 19.60±6.32 [18.03–21.16]) than that in the LC, LC + Gaussian, LC + NLM, and reference images (mean±SD [95% CI], 24.33±5.33 [23.00–25.65], 22.22±5.55 [20.84–23.60], 21.24±5.78 [19.81–22.67] and 21.88±6.33 [22.28–25.33], respectively). Figure 7 shows the CV of the background fibroglandular tissue or fat tissue at the edge of the FOV.

 Quantitative assessment was performed for a total of twenty-two lesions in the test data set. The details of the lesions are listed in Table 2. Six lesions were focus lesions and sixteen were mass uptake lesions.

**Figure 8:**
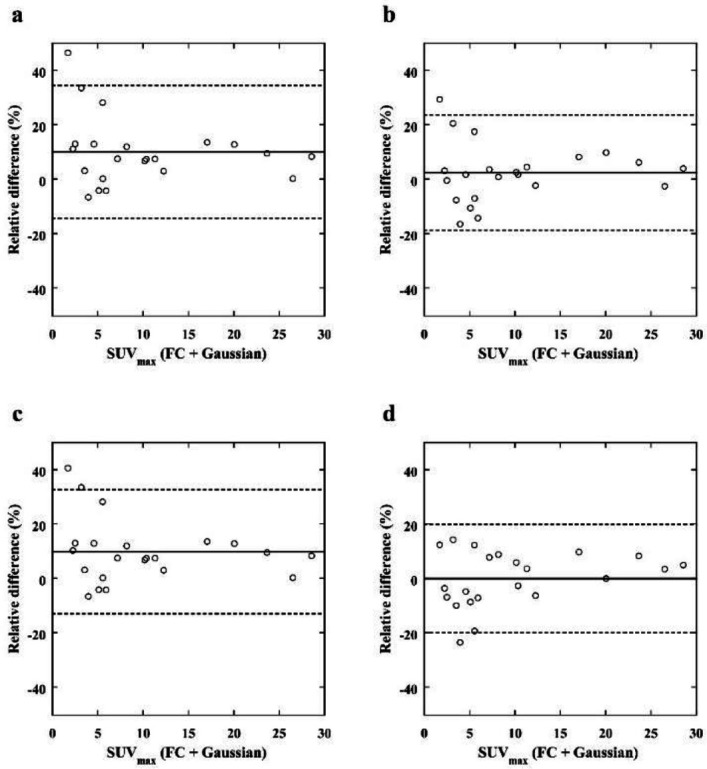
Bland– Altman plot of the relative differences in the SUV_max_ of breast lesions between the reference (FC + Gaussian) images and the LC (**a**), LC + Gaussian (**b**), LC + NLM (**c**), and LC + DL (**d**) target images. The solid and dashed lines indicate the mean bias and variance, respectively. The bias and variance of the relative differences in the LC + DL images are smaller than those of the other images

**Figure 9:**
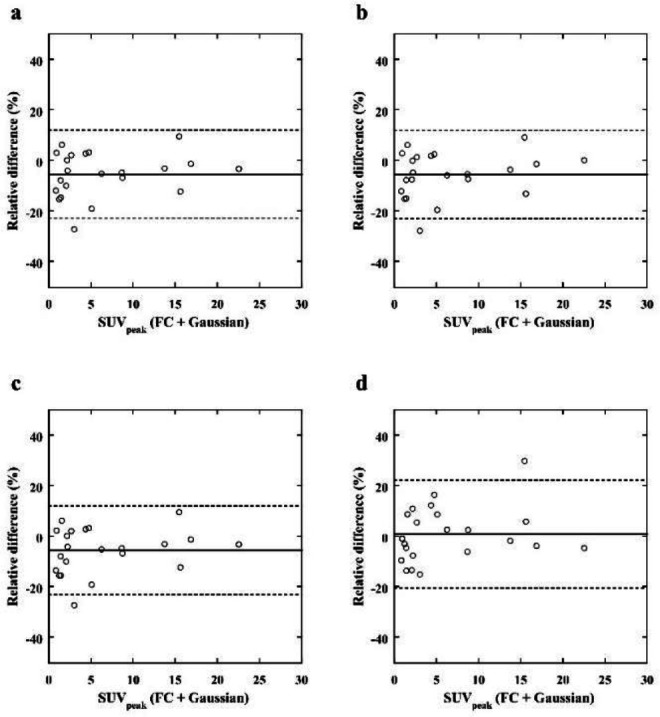
Bland– Altman plot of the relative differences in the SUV_peak_ of breast lesions between the reference (FC + Gaussian) images and the LC (**a**), LC + Gaussian (**b**), LC + NLM (**c**), and LC + DL (**d**) target images. The solid and dashed lines indicate the mean bias and variance, respectively. The bias of the relative differences in the LC + DL images is smaller than those of the other images

 The SUV_max_ of the lesions for the LC (median, 2.48; interquartile range (IQR), 4.39 to 12.51) and LC + NLM (median, 2.39; IQR, 4.39 to 12.51) images were significantly higher than those for

the reference images (median, 1.70; IQR, 4.11 to 12.02) (*p*<0.001, *p*<0.001, respectively), and no significant differences in SUV_max_ were observed for the LC + Gaussian (median, 2.20; IQR, 4.00 to 11.93) and LC + DL images (median, 1.91; IQR, 3.80 to 11.65) when compared with the reference images. The SUV_peak_ for the LC (median, 0.74; IQR, 1.69 to 8.22), LC + Gaussian (median, 0.73; IQR, 1.71 to 8.17), and LC + NLM (median, 0.72; IQR, 1.69 to 8.22) images were significantly higher than those for the reference images (median, 0.84; IQR, 1.67 to 8.72) (*p*<0.033, *p*<0.042, *p*<0.033, respectively), and no significant differences in SUV_peak_ were observed for the LC + DL images (median, 0.76; IQR, 1.70 to 8.75) when compared with the reference images. Table3 shows the results of the SUV_max_ and SUV_peak_ of the lesions. The relative differences for SUV_max_ and SUV_peak _are shown in Bland– Altman plots in Figure 8 and Figure 9, respectively. The Bland–Altman plots show the lowest mean bias of the relative differences for SUV_max_ and SUV_peak_ (−0.07 % and 0.80 %) in the LC + DL images. In the LC + DL images, the variance of the relative difference in SUV_max_ was the smallest, while that for SUV_peak_ was the largest.

**Table 2:** Characteristics of the 22 lesions evaluated in the test data set

**Characteristics**	**Number ** **(** **%) or value**
**Diagnosis**	
Invasive ductal carcinoma*	14 (64.6)
Ductal carcinoma in situ*	6 (27.3)
Paget disease*	1 (4.5)
Lymph node metastasis**	1 (4.5)
**U** **ptake type**	
Focus	6 (27.3)
Mass uptake	16 (72.7)
Non- mass uptake	0 (0)
**S** **UV**_max_ ** (mean±SD** **)** * * *	
All	9.95±8.15
Focus	2.84±0.85
Mass uptake	12.61±8.05

* Diagnosed based on the histopathological findings

** Diagnosed based on imaging studies

*** SUV_max_ measured on the reference (FC + Gaussian) images

**Table 3:** The results of the SUV_max_ and SUV_peak_ of the lesions

**Image sets**	**Median (Interquartile range)** **SUV**_max _ **SUV**_peak_	
FC + Gaussian	1.70 (4.11-12.02)	0.84 (1.67-8.72)
LC	2.48 (4.39-12.51)	0.74 (1.69-8.22)
LC + Gaussian	2.20 (4.00-11.93)	0.73 (1.71-8.17)
LC+NML	2.39 (4.39-12.51)	0.74 (1.69-8.22)
LC + DL	1.91 (3.80-11.65)	0.76 (1.70-8.75)

Data are presented as median and interquartile range

Table 4 and 5 shows the results of the visual evaluation of smoothness and contrast for all image sets, respectively. The smoothness for the LC + DL images was significantly better than that for the other images except for the reference images (*p*<0.001). Furthermore, the smoothness for the reference images was significantly better than that for the other images except for the LC + DL images (*p*<0.001). The smoothness for the LC + NLM images was also significantly better than that for the LC + Gaussian images (*p*=0.003). No significant differences in the lesion contrast were observed in all image sets.

**Table 4:** Smoothness evaluated by visual assessment

**Visual score**					
	**FC + Gaussian**	**LC**	**LC + Gaussian**	**LC+NML**	**LC + DL**
0	8 (6.7)	16 (13.3)	14 (11.7)	22 (18.3)	4 (3.3)
1	20 (16.7)	83 (69.2)	77 (64.2)	81 (67.5)	37 (30.8)
2	87 (72.5)	21 (17.5)	29 (24.2)	17 (14.2)	73 (60.8)
3	5 (4.2)	0 (0)	0 (0)	0 (0)	6 (5.0)
kappa	0.15	0.20	0.22	0.18	0.29

Data are presented as number (%)

**Table 5:** Lesion contrast evaluated by visual assessment

**Visual score**					
	**FC + Gaussian**	**LC**	**LC + Gaussian**	**LC+NML**	**LC + DL**
0	1 (2.3)	0 (0)	0 (0)	0 (0)	0 (0)
1	1 (2.3)	1 (2.3)	1 (2.3)	1 (2.3)	2 (4.5)
2	1 (2.3)	2 (4.5)	2 (4.5)	2 (4.5)	1 (2.3)
3	41 (93.2)	41 (93.2)	41 (93.2)	41 (93.2)	41 (93.2)
kappa	0.31	0.31	0.31	0.31	0.65

Data are presented as number (%)

## Discussion

 The present study showed that the quantitative values of lesions could be preserved at less than half of the emission time when using the Res-Net model to reduce noise.

 In [^18^F]FDG PET examinations, it is desirable to reduce the acquisition time or injected activity; however, an insufficient count will lead to an increase in image noise. The noise can influence diagnosis, decrease the detectability of small lesions, and affect the SUV measurement. In the present study, our model significantly reduced noise due to reduced acquisition time when compared with the use of a Gaussian and an NLM filter. A post-smoothing filter is usually adopted to reduce noise in PET images, but its performance is limited because it is designed to reduce Gaussian random noise, which is distinct from the noise in PET images (36, 37). Our model can effectively reduce the noise in PET images, which is characterized by a complex noise distribution. An NLM filter is mainly used to remove Gaussian noise and speckle noise. In addition, the use of this filter requires the standard deviation of the noise to be set, and an improper setting will lead to blur in the images (29). The dbPET system has the characteristic of low sensitivity at the edge of the detector (17), and noise distributions in the trans-axial images used as the input of our model vary with respect to the location in the plane. Therefore, we believe it is challenging to determine the optimal parameters for the NLM filter. The present study showed that our model, which uses a larger filter size than the model in a previous report (30), was able to capture more context information in a larger image region and efficiently reduce the levels of location-dependent noise (38, 39).

 Our model slightly changed the SUV_mean_ of the background fibroglandular tissue compared to the reference images, as well as the use of the NLM filter. Non-linear image processing (e.g., the NLM filter or deep learning based denoising) may result in a slight shift the mean value of the image, but we consider this to have minimal clinical impact.

 Semiquantitative analysis using the SUV is used to diagnose malignancy as well as monitor the response to therapy of a breast tumor (2). In the present study, our model obtained the lowest bias of the relative differences for SUV_max_ and SUV_peak_. We believe that noise reduction using our model removed the variability in quantitative values of the lesions, while maintaining the SUV_mean_ of the background fibroglandular tissue. These results are consistent with those of a previous study on noise reduction for low-dose [^18^F]FDG PET images using a supervised deep learning model (40). Using our model, the variance of the relative difference for SUV_max_ was the smallest, whereas that for SUV_peak_ was the largest. The SUV_peak_ was measured in an ROI centered at the maximum value of the lesions; therefore, the position of the ROI was not always identical among the images. In some cases, noise reduction with our model may have caused the position of the ROI to change with respect to the ROI in the reference images, which also has a degree of noise, resulting in an increase in the variability of the relative difference in SUV_peak_.

 A Gaussian filter leads to a slight decrease in SUV_max_ of lesions due to blurring. In this study, our model was trained using the image sets without a Gaussian filter, but denoised images obtained using our model exhibited comparable quantitative values to the reference images with a Gaussian filter. The network used in this study was trained with the MSE as a loss function, which is known to introduce slight blurring in the network output (25). In medical imaging, there are some reports that using the structural similarity index and perceptual loss as a loss function may improve the result and should be considered in future (41- 43).

 The model used in this study consisted of fewer layers, whereas the filter size was larger than that used in a prior study (30), resulting in increased computational cost. The use of dilated convolution is expected to improve efficiency while maintaining performance (38).

 There were several limitations in this study. First, the detectability of small lesions was not evaluated in this study. In general, the choice of a post-smoothing filter is concerned about loss of details in tumor structure and the reduced detectability of small lesions. Furthermore, there are some reports that an NLM filter leads to blurring and loss of the details of high-contrast small lesions especially in images with high noise levels (44, 45). Because PET images tend to have more noise in regions of high uptake (46), an NLM filter may cause reduced detectability of small lesions in the higher accumulation of the background fibroglandular tissue. Therefore, we believe it is important to compare the detectability of small lesions in images using our model and other post-smoothing filters. However, the visualization of the six focus lesions were maintained in our study. Furthermore, there were no non-mass uptake lesions in the test data, and future studies are needed to assess the influence of our 

denoising model on non-mass uptake lesions. Second, our sample size was relatively small. Only twenty-eight patients were included in this study population and there were only twenty-two lesions. Data augmentation is one way to increase training data, but excessive data augmentation can lead to unpredictable results. Moreover, the noise distributions in the dbPET images vary with respect to the location in the plane. A post-smoothing filter denoise the image uniformly, but our model could have been efficiently reducing the location-dependent noise, especially on the chest wall side. However, the amount or distribution of the noise on the chest wall side varies among patients, so it takes a large number of sample size to investigate the detectability of lesions on the chest wall side. Thus, a larger sample size is required to create models and evaluate the detection performance. In addition, further consideration should be given to how the network is trained, particularly with respect to input images for the network. 2D images were used as the input of the network in this study, but previous reports have shown good performance of PET image denoising using 3D or 2.5D images as the inputs of the network (27, 28). The use of 3D or 2.5D images as the inputs may be possible to improve image quality and should be investigated in the future. Furthermore, we did not eliminate slices that include only air in this study, and it may be possible to improve the result by using only the slices include the breast.

 The present study reduced the noise from dbPET images obtained in about half the emission time, and further reduction of emission time could be possible by training or evaluating models with fewer counts images.

## Conclusion

 The present study showed that the use of the Res-Net model reduced the noise in dbPET images acquired in about half the emission time while preserving quantitative values of lesions. The machine learning is feasible in the noise reduction in dbPET images and potentially performs better than conventional post-image filtering.

## Competing interests

 Author Masakazu Toi has received research support from Shimadzu Corporation.

## Funding

 No funding was received for conducting this study.
